# Enhancing Immune Responses to Cancer Vaccines Using Multi-Site Injections

**DOI:** 10.1038/s41598-017-08665-9

**Published:** 2017-08-16

**Authors:** Robert C. Mould, Amanda W. K. AuYeung, Jacob P. van Vloten, Leonardo Susta, Anthony J. Mutsaers, James J. Petrik, Geoffrey A. Wood, Sarah K. Wootton, Khalil Karimi, Byram W. Bridle

**Affiliations:** 1 Department of Pathobiology, Ontario Veterinary College, University of Guelph, Guelph, Ontario, N1G 2W1 Canada; 2 Department of Biomedical Sciences, Ontario Veterinary College, University of Guelph, Guelph, Ontario, N1G 2W1 Canada; 3 Department of Clinical Studies, Ontario Veterinary College, University of Guelph, Guelph, Ontario, N1G 2W1 Canada

## Abstract

For a vaccine to be effective it must induce a sufficiently robust and specific immune response. Multi-site injection protocols can increase the titers of rabies virus-neutralizing antibodies. Hypothetically, spreading a vaccine dose across multiple lymphatic drainage regions could also potentiate T cell responses. We used a replication-deficient adenovirus serotype 5-vectored cancer vaccine targeting the melanoma-associated antigen dopachrome tautomerase. Clinically, high numbers of tumor-infiltrating CD8^+^ T cells are a positive prognostic indicator. As such, there is interest in maximizing tumor-specific T cell responses. Our findings confirm a positive correlation between the number of tumor-specific T cells and survival. More importantly, we show for the first time that using multiple injection sites could increase the number of vaccine-induced CD8^+^ T cells specific for a self-tumor antigen. Further, the number of tumor antigen-specific antibodies, as well CD8^+^ T cells specific for a foreign antigen could also be enhanced. Our results show that multi-site vaccination induces higher magnitude immune responses than a single-bolus injection. This provides a very simple and almost cost-free strategy to potentially improve the efficacy of any current and future vaccine. Broader clinical adoption of multi-site vaccination protocols for the treatment of cancers and infectious diseases should be given serious consideration.

## Introduction

Developing cancer vaccines is an emphasis in the field of immunotherapy^1^. Although all immunological effector mechanisms can contribute to regression of cancers, there is a focus on inducing tumor-specific T cells due to their natural, dominant role in cancer immunosurveillance^2^. Indeed, the number of effector T cells infiltrating a patient’s tumor often correlates with clinical outcomes such as progression-free and overall survival^3^. Therefore, a variety of strategies are being investigated to increase the number of vaccine-induced T cells. Clinically, vaccines are typically administered as single-bolus injections. However, this will drive antigen presentation in a very limited number of draining lymph nodes. Interestingly, the World Health Organization encourages injection of rabies vaccines into multiple sites around the body to induce higher titers of virus-neutralizing antibodies for post-exposure prophylaxis^4, 5^. Despite evidence that multi-site vaccination was superior to single-bolus injections for inducing rabies-specific antibodies, this strategy has not been broadly implemented with other vaccines. This may be due to concerns over using multiple needle punctures in patients, especially if single-bolus injections are usually protective, can be boosted later with single-bolus secondary vaccines or the disease is relatively minor. However, cancers represent a fundamentally different scenario. Although cancer vaccines can extend survival, most patients eventually succumb to the disease^6^. Also, most cancer vaccines need to be administered in a therapeutic setting where there is rapidly increasing numbers of target cells with a concomitant increase in immunosuppressive potential. This is further confounded by the fact that many tumor-associated antigens that have been identified as reasonable targets are derived from self^7^. This means that vaccines need to engage a very limited repertoire of autoreactive T cells that have survived thymic selection. In this context, we postulate that cancers are an ideal setting for implementation of multi-site vaccination to potentiate tumor-specific immune responses by maximizing the engagement of secondary lymphoid tissues. Here we provide evidence for the first time that increasing the number of injection sites for a cancer vaccine correlates with higher magnitude tumour-specific T cell and antibody responses.

## Results and Discussion

**Figure 1:**
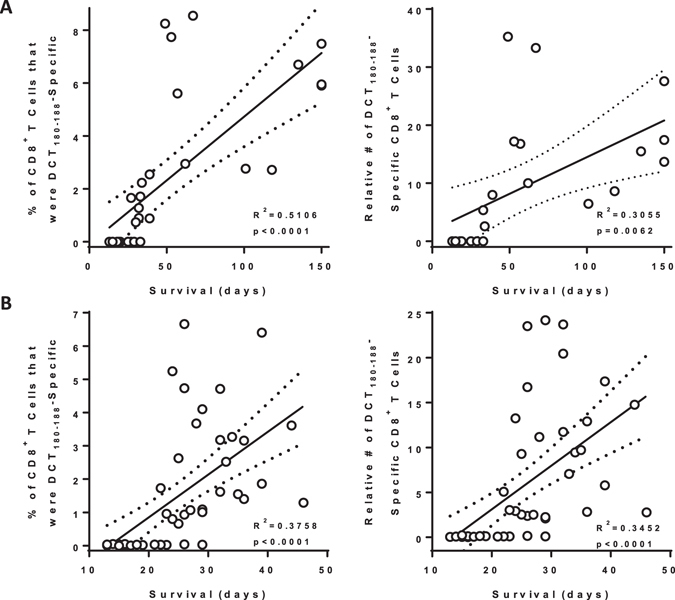
Survival correlated with the magnitude of antigen-specific blood-derived CD8^+^ T-cell responses in prophylactic and therapeutic models of metastatic brain cancer. Female C57BL/6 mice had 1,000 B16-F10 melanoma cells stereotactically implanted into the parenchyma of the right hemisphere of the brain. (**A**) Eleven days prior to, or (**B**) five days after intracranial challenge, mice were vaccinated intramuscularly with 1 × 10^8^ pfu of recombinant human serotype 5 adenovirus expressing the melanoma-associated antigen dopachrome tautomerase, with injections administered into the semitendinosus of both hind limbs. Blood-derived CD8^+^ T cells specific for the immunodominant self-epitope DCT_180–188_ were quantified fourteen days post-vaccination by flow cytometric assessment of intracellular cytokine staining after *ex vivo* re-stimulation with peptides (supplementary Figure 1). Pearson correlation analysis was performed for overall survival versus frequency (left panel; *n* = 33 for [A] and 52 for [B]) and total number (right panel; *n* = 23 for [A] and 52 for [B]; pooled from five and ten experimental replicates, respectively) of antigen-specific CD8^+^ T cells. Lines of best fit with 95% confidence intervals are shown.

Historically, our research group operated under the assumption that single-bolus injections would engage a limited amount of lymphatic tissues proximal to the vaccination site. This would leave many potential lymphatic draining regions naïve to the vaccine, causing sub-optimal immune responses. Therefore, we always delivered pre-clinical cancer vaccines intramuscularly into both hind limbs of mice instead of as a single bolus. This also assumed that higher magnitude tumor-specific immune responses would translate into better efficacy. However, we never formally tested either of these hypotheses. Therefore, we selected a vaccine with which we have experience; a recombinant, E1/E3-deleted, replication-deficient human serotype-5 adenovirus (Ad5) encoding human dopachrome tautomerase (DCT), which is a melanoma-associated antigen^8^. We leveraged the relatively broad range in the magnitude of immune responses that could be generated in a large number of mice using the two-site injection protocol to determine if T cell numbers correlated with survival in a metastatic model of B16-F10 melanoma in the brain^9^. The dominant effectors in this model were previously shown to be CD8^+^ T cells^9^. Mice (C57BL/6; H-2K^b^ haplotype) were vaccinated with 1 × 10^8^ pfu of Ad5-DCT into the semitendinosus muscles of both hind limbs, followed eleven days later with intracranial injection of 1 × 10^3^ B16-F10 cells. Eleven days post-vaccination (peak of the primary response), blood-derived CD8^+^ T cells specific for the immunodominant self-epitope DCT_180-188_ were quantified. In this prophylactic scenario, both frequency and number of DCT-specific CD8^+^ T cells correlated with overall survival (Fig. 1a, left panel, R^2^ = 0.5106 [p < 0.0001] and right panel, R^2^ = 0.3055 [p = 0.0062], respectively). Prophylactic vaccines represent a small portion of novel immunotherapies progressing through clinical trials since the onset of most cancers cannot be predicted. Therefore, we sought to see if this principle remained true in a therapeutic setting, where an established tumor can suppress immune responses^10^. Mice were challenged intracranially with B16-F10 cells five days before vaccinating with Ad5-DCT into the muscles of both hind limbs. Again, overall survival correlated with the frequency and number of DCT-specific T cells circulating in blood (Fig. 1b, left panel, R^2^ = 0.3758 [p < 0.0001] and right panel, R^2^ = 0.3452 [p < 0.0001], respectively). These findings confirm that maximizing cancer-specific T cell responses can translate into increased survival.

**Figure 2:**
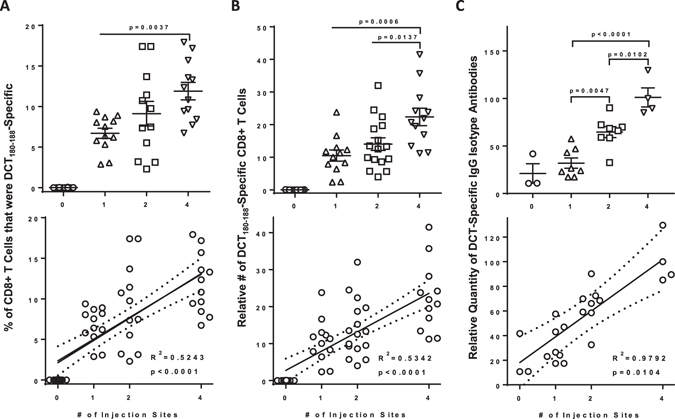
The magnitude of blood-derived antigen-specific CD8^+^ T cell and antibody responses correlated with the number of vaccine injection sites. Tumor-free female C57BL/6 mice were vaccinated intramuscularly with 1 × 10^8^ pfu of recombinant human serotype 5 adenovirus expressing the melanoma-associated antigen dopachrome tautomerase, with injections spread across one (semitendinosus; left hind limb), two (semitendinosus of both hind limbs) or four (semitendinosus of both hind limbs and the triceps brachii of both forelimbs) sites. (**A**,**B**) Blood-derived CD8^+^ T cells specific for the immunodomiant self-epitope DCT_180–188_ were quantified fourteen days post-vaccination by flow cytometric assessment of intracellular cytokine staining after *ex vivo* re-stimulation with peptides. One-way analysis of variance with Tukey’s multiple comparison test (upper panels) and Pearson correlation analysis (lower panels) was performed for the (**A**) frequency and (**B**) total number of antigen-specific CD8^+^ T cells (*n* = 12–16/treatment; pooled from three experimental replicates). (**C**) Plasma-derived antibodies (IgG) specific for dopachrome tautomerase were quantified thirty-three days post-vaccination using an in-cell western blotting assay and assessed by one-way analysis of variance (upper panel) and Pearson correlation analysis (lower panel; *n* = 3 controls and 4–8/treatment; pooled from two experimental replicates). Correlation analyses show lines of best fit with 95% confidence intervals.

Knowing that Ad5-DCT-induced immune responses in blood correlated with survival, we focused on testing the hypothesis that multi-site vaccination could induce more transgene-specific T cells than a single-bolus delivery. If true, it would suggest that increasing the number of vaccination sites beyond two may lead to even higher magnitude immune responses. Tumor-free mice were vaccinated with a fixed dose of 1 × 10^8^ pfu of Ad5-DCT injected into one (semitendinosus of the left hind limb), two (both hind limbs), or four (semitendinosus of both hind limbs and triceps brachii of both forelimbs) discrete lymphatic drainage regions. We assessed the magnitude of the CD8^+^ DCT_180-188_-specific T cell response in blood eleven days post-vaccination. As hypothesized, multi-site vaccination was superior to single-bolus injections with respect to inducing higher frequencies (Fig. 2a, R^2^ = 0.5243 [p < 0.0001]) and numbers (Fig. 2b, R^2^ = 0.534 [p < 0.0001]) of tumor-specific T cells. As compared to a single-bolus injection, T cell responses were doubled when the same vaccine dose was spread across four sites. Since the inclusion of the triceps brachii was unique to the four-site vaccination group, we investigated whether there was an anatomical advantage to vaccinating the fore- versus hind-limbs. Notably, there was no significant advantage to injecting the vaccine into both triceps brachii as opposed to the semitendinosus muscles of both hind legs (supplementary Figure 2). Therefore, we concluded that the higher magnitude CD8^+^ T cell responses in the four-site injection group was likely due to engagement of a greater number of lymph nodes and not due to an inherent advantage of targeting secondary lymphoid tissues that survey the forelimbs. Further, the benefit of multi-site injections was replicated in tumor-bearing hosts. To test this, we challenged mice with intradermal injections of 2 × 10^5^ B16-F10 cells, followed four days later by vaccination with Ad5-DCT at one, two or four sites. Blood-derived DCT-specific CD8^+^ T cells were most numerous in mice receiving multi-site injections (supplementary Figure 3), suggesting that this method is also of benefit in the immunosuppressive environment of a tumor-bearing host.

Our inspiration to test multi-site vaccination to enhance tumor-specific T cell responses was the previous application to increase rabies virus-specific antibody titers^4, 5^. Tumor-specific antibodies can target antigens expressed on the surface of cancer cells. However, even antibodies against intracellular antigens such as DCT can contribute to killing tumor cells^11^, presumably by promoting uptake of target proteins by antigen-presenting cells and/or facilitating antibody-dependent cell-mediated cytotoxicity^12^. We previously showed that Ad5-DCT could induce DCT-specific IgG antibodies^13^. To determine if multi-site vaccination could potentiate this antibody response, we quantified DCT-specific antibodies in plasma 33 days after tumour-free mice had received 1 × 10^8^ pfu of Ad5-DCT injected in one, two or four sites. The number of injection sites had a strong positive correlation with the concentration of DCT-specific IgG antibodies in blood (Fig. 2c, R^2^ = 0.9541 [p < 0.0232]). This extends the previous discovery that multi-site vaccination can increase virus-specific antibody titers^4, 5^ to antibodies targeting tumor-associated antigens. Therefore, multi-site vaccination has utility for induction of tumor-specific T and B cell responses.

**Figure 3:**
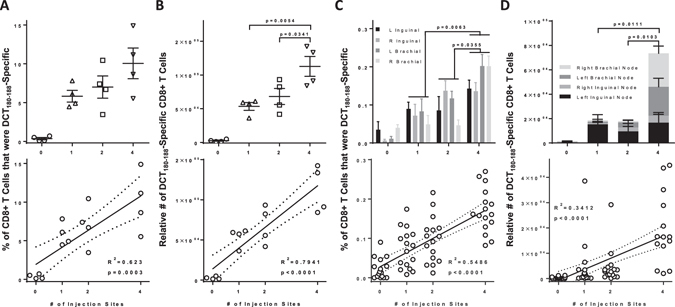
Multi-site vaccination increased the magnitude of transgene-specific CD8^+^ T cells in spleens and lymph nodes. Tumor-free female C57BL/6 mice were vaccinated intramuscularly with 1 × 10^8^ pfu of recombinant human serotype 5 adenovirus expressing the melanoma-associated antigen dopachrome tautomerase, with injections spread across one (semitendinosus; left hind limb), two (semitendinosus of both hind limbs) or four (semitendinosus of both hind limbs and the triceps brachii of both forelimbs) sites. (**A**,**B**) Spleen and (**C**,**D**) lymph node-derived CD8^+^ T cells specific for the immunodominant self-epitope DCT_180–188_ were quantified fourteen days post-vaccination by flow cytometric assessment of intracellular cytokine staining after *ex vivo* re-stimulation with peptides. (**A**,**B**) One-way analysis of variance with Tukey’s multiple comparison test (upper panels) and Pearson correlation analysis (lower panels) was performed for the (**A**) frequency and (**B**) total number of antigen-specific splenic CD8^+^ T cells (*n* = 4/treatment). (**C–D**) Two-way analysis of variance with Tukey’s multiple comparison test (upper panels) and Pearson correlation analysis (lower panels) was performed for the (**C**) frequency and (**D**) total number of antigen-specific lymph node-derived CD8^+^ T cells (*n* = 4/treatment; L = left node, R = right node).

To this point, results showed that increasing the number of vaccine injection sites could increase the number of circulating tumor-specific T cells. However, blood contains only a small proportion (~2%) of the total lymphocytes in the body^14^. To assess whether multi-site vaccination could globally increase responses, we immunized tumor-free mice with Ad5-DCT at one, two or four sites and harvested spleens and dominant regional draining lymph nodes (*i.e*. inguinal and brachial^15^). Similar to blood, the number of injection sites had a strong positive correlation with the frequency and number of splenic DCT-specific CD8^+^ T cells (Fig. 3a, R^2^ = 0.6230 [p = 0.0003] and 3b, R^2^ = 0.7941 [p < 0.0001], respectively). Positive correlations were also observed with the number of vaccination sites and the total frequency and number of transgene-specific CD8^+^ T cells when data from all four lymph nodes (*i.e*. both inguinal and both brachial) were combined (Fig. 3c, R^2^ = 0.5486 [p < 0.0001] and 3d, R^2^ = 0.3412 [p < 0.0001], respectively). As expected, T cell responses were greatest in lymph nodes that drained vaccination sites. Taken together these data suggest a global increase in T cell responses when using multi-site vaccination.

Autoreactive T cells are typically present at low frequencies. Maximizing the amount of secondary lymphoid tissue involved in presenting antigens to rare T cell populations would seem to be a logical way to optimize their numbers. However, we also wanted to determine if the benefit of multi-site vaccination would be observed in the context of T cells specific for a foreign antigen that should exist in higher numbers in the body due to less rigorous thymic selection. Tumor-free mice were vaccinated with Ad5 expressing the immunodominant CD8^+^ T cell epitope of chicken ovalbumin (OVA_257–264_) in one, two or four sites. Although the multi-site vaccination protocol did not significantly increase the frequency of blood-derived OVA_257–264_-specific T cells, the absolute number was almost doubled in mice that had been vaccinated at four sites (supplementary Figure 4). This suggests that T cell responses against foreign antigens may not be as sensitive to the benefits of multi-site vaccination as compared to self-antigen-specific T cells. Nonetheless, our data support the use of a four-site injection protocol to maximize the induction of T cell responses to both self-derived and foreign antigens. This further extends the previous results with rabies vaccines^4, 5^, to provide evidence that multi-site vaccination could benefit treatment of infectious diseases not only by enhancing antibody titers, but also T cell responses.

The World Health Organization’s data from rabies immunizations show that multi-site vaccination can enhance antibody responses to foreign antigens^4, 5^. Our results extend this observation to a tumor-associated antigen and demonstrate, for the first time, that engaging multiple distinct lymphatic regions with a vaccine can also potentiate T cell responses against both foreign and self-antigens. Overall, the evidence argues that multi-site vaccination is superior to single-bolus injections when aiming to maximize cell- and antibody-mediated immune responses to foreign and self-antigens in healthy and tumor-bearing hosts. This has important implications for treating infectious diseases and cancers with vaccines. We tested this approach using a maximum of four injection sites. It may be possible to derive additional benefit by further increasing the number of sites. A report on rabies vaccines suggested that eight-site administration was not better than four sites for antibody responses^4^. However, this may not be the case for T cell responses against foreign antigens nor for antibody and T cell responses against self-antigens. Indeed, our results suggested that multi-site vaccination may be most beneficial in the induction of responses against a self-antigen. Testing a four- versus eight-site immunization protocol with a cancer vaccine would be a logical extension of this research. Another interesting question that arises from this research is whether the benefits of multi-site vaccination can be extended to non-viral-vectored vaccines (*e.g*. DNA-, peptide-, dendritic cell-based, *etc*.) We acknowledge that for some vaccines, patient adversity to multiple needle punctures may preclude consideration of a multi-site protocol for diseases that are currently well-controlled. However, for potentially fatal conditions like cancers, where induction of maximal immune responses are warranted, clinicians should give serious consideration to administering vaccines into multiple distinct lymphatic drainage regions such as both upper arm/deltoid regions and both upper leg/gluteal regions. Multi-site vaccination represents a simple, almost cost-free strategy for improving immune responses that can be applied to any existing and future vaccines.

## Methods

### Mice

Female specific pathogen-free C57BL/6 mice (major histocompatibility haplotype b; Charles River Laboratories, USA), at 36–50 days of age upon arrival, were housed in a controlled environment the Isolation Unit at the University of Guelph (Guelph, Ontario, Canada). Food and water were provided *ad libitum*. Mice were acclimatized to the facility for one week prior to beginning experiments. Animal studies complied with Canadian Council on Animal Care guidelines and were approved by the Animal Care Committee (University of Guelph; animal utilization protocol #1904).

### Viruses

Replication-deficient E1/E3-deleted, recombinant human adenovirus serotype 5 (Ad5), expressing the melanoma-associated antigen human dopachrome tautomerase (DCT) has been described previously and was propagated in human embryonic kidney-293 cells (American Type Culture Collection, USA) and purified by ultracentrifugation on a cesium chloride gradient^16, 17^. A second Ad5, expressing the immunodominant epitope (for H-2K^b^) from chicken ovalbumin (OVA_257–264_; SIINFEKL) and firefly luciferase was used and has also been described^18^. Replication-competent recombinant vesicular stomatitis virus (Δm51 mutant of the Indiana serotype) carrying a transgene encoding human DCT was used to infect Vero cells (American Type Culture Collection [ATCC], USA) as part of an in-cell western blotting assay to quantify DCT-antibodies. VSVΔm51-hDCT has been described previously and was propagated and titered in Vero cells^19, 20^.

### Cell Cultures

Human embryonic kidney-293 cells (ATCC) were cultured in Eagle’s Minimum Essential Medium containing 10% heat-inactivated bovine calf serum (GE Healthcare Life Sciences, USA). Murine B16-F10 melanoma cells (ATCC) that were used to establish tumors were cultured in Dulbecco’s Modified Eagle’s Medium (GE Healthcare Life Sciences) containing 10% bovine calf serum. Vero cells were used for an in-cell western blotting assay to quantify antibody responses. They were also grown in Dulbecco’s Modified Eagle’s Medium with 10% bovine calf serum. All cells were cultured at 37 °C in a humidified atmosphere with 5% CO_2_ and were confirmed to be mycoplasma-free prior to use (MycoAlert PLUS detection kit, Lonza, USA).

### Model of Metastatic Cancer in the Brain

As reported previously^9^, melanomas were established in the brains of mice by intracranial injections of 1,000 B16-F10 cells in 2 μl of phosphate buffered saline (PBS; GE Heathcare). Specifically, mice were placed in a stereotaxis (Xymotech Biosystems, Mt. Royal, Quebec, Canada) and an incision was made in the scalp to expose the skull under both general and local anesthesia. A 25-gauge needle mounted on a 10 μl Hamilton syringe (Hamilton, Reno, NV, USA) was positioned over the right hemisphere of the brain, 2.25 mm lateral to Bregma. A small hole was drilled through the skull and the bevel of the needle inserted into the brain parenchyma to a depth of 3 mm. Cells were injected over a period of 2 minutes. The needle was left in place for 2 minutes prior to withdrawal to minimize reflux along the injection tract. The scalp incision was closed with stainless steel clips that were removed seven days later.

### Vaccination Protocol

All vaccinated mice were given a total dose of 1 × 10^8^ pfu of Ad5 via intramuscular injection. For one-site vaccination groups, 1 × 10^8^ pfu of Ad5 was given as a single-bolus injection into the semitendinosus of the left hind limb in 50 μl of PBS. For two-site vaccination groups, 5 × 10^7^ pfu of Ad5 in 50 μl of PBS was injected into the semitendinosus of both hind limbs. Four-site vaccination groups received 2.5 × 10^7^ pfu of Ad5 in 50 μl of PBS in the semitendinosus of both hind limbs and the triceps brachii of both forelimbs. Control groups were not vaccinated.

### Multi-Site Injections in Skin Melanoma-Bearing Hosts

C57BL/6 mice were challenged intradermally with 2.5 × 10^5^ B16-F10 melanoma cells. Four days later, mice were randomly assigned to four treatment groups (*n* = 4 per group). Mice were vaccinated with 1 × 10^8^ pfu of Ad5-DCT at one, two or four sites; controls were unvaccinated. Antigen-specific T cells were quantified at the peak of the primary response (*i.e*. fourteen days post-vaccination).

### Sample Processing

Blood samples from the retro-orbital sinus were heparinized to prevent clotting. Blood volumes were recorded and then blood was centrifuged to collect and archive plasma for quantification of antibodies. Spleens and lymph nodes (inguinal and brachial) were harvested in Hanks’ Balanced Salt Solution (HBSS; GE Healthcare Life Sciences). Samples were kept on ice during transportation and processing. Single-cell suspensions were made by pressing spleens and lymph nodes between the frosted ends of glass slides. Erythrocytes in blood and spleens were lysed. Cells were counted using an improved Neubauer counting chamber using trypan blue dye exculsion to assess viability, which was consistently >90%. All cells were plated in RPMI-1640 medium containing 10% heat-inactivated bovine calf serum and penicillin/streptomycin (cRPMI; GE Healthcare Life Sciences) in 96-well round-bottom plates and were maintained at 4 °C during handling and centrifugation (500 g). Up to two million cells were added to each well. Samples were processed by the same individual to reduce technical variability and they were blinded to treatment groups to eliminate bias.

### Quantification of T Cells by Flow Cytometry

Blood-, spleen and lymph node-derived cells were re-stimulated by adding transgene-derived peptides to a final concentration of one µg/mL. Controls were unstimulated. After one hour, brefeldin A (ThermoFisher Scientific, USA) was added to each sample to block golgi apparatus-mediated export of proteins. After another four hours, cells were centrifuged, re-suspended in PBS + 1% bovine serum albumin and stained with antibodies. Fc receptors were blocked with anti-CD16/CD32 (clone 93; BD Biosciences, USA) for fifteen minutes. Surface staining for 20 minutes was performed with anti-CD3 and anti-CD8 (clones 145-2C11 and 53–6.7, respectively; BD Biosciences). A fixable viability dye stain (ThermoFisher Scientific) was used to ensure that only live cells would be included in analyses. Following treatment with Intracellular Fixation Solution (ThermoFisher Scientific) for 20 minutes, intracellular staining was done for 20 minutes using anti-TNF-α and anti-IFN-γ in a permeabilization solution (clones MP6-XT22 and XMG1.2, respectively; ThermoFisher Scientific). Stained cells were suspended in 200 μl of PBS + 1% bovine serum albumin, filtered through a mesh with 70 μm pores and analyzed using a FACS Canto II flow cytometer (Becton Dickinson) using FACS Diva Software Version 8 for data acquisition. Analysis was completed with FlowJo Software version 10 (FlowJo LLC, Ashland, Oregon, USA). An example of the gating strategy used to quantify antigen-specific CD8^+^ T cells is shown in supplementary Figure 1.

### Peptides

For quantification of DCT-specific CD8^+^ T cell responses, cells were re-stimulated *ex vivo* with the immunodominant peptide that binds to H-2K^b^ (DCT_180–188_; SVYDFFVWL, which is conserved in human and murine DCT; PepScan Systems, Lelystad, Netherlands). Similarly, for characterization of ovalbumin (OVA) specific responses, the H-2K^b^-restricted immunodominant epitope OVA_257–264_ was used (SIINFEKL; PepScan Systems).

### Quantification of Antibody Responses by In-Cell Western Blotting

Confluent monolayers of Vero cells in flat-bottom 96-well plates were infected with VSVΔm51-hDCT at a multiplicity of infection of 10 and incubated for 12 hours. Cells were washed with HBSS, fixed in 3.7% paraformaldehyde and permeabilized with 0.2% Triton-X100. Plates were washed and blocked with 50 μL per well of PBS + 1% bovine serum albumin (BSA) for 30 minutes at room temperature. Plasma from mice was thawed, diluted 1/200 in cold PBS + 1% BSA and added to fixed cells (100 μL per well) for one hour at room temperature. Samples were tested in duplicate. Plates were washed with PBS + 1% BSA and 50 μL per well of 1:2,000 goat anti-mouse IgG (H + L) conjugated to alexa fluor-488 (Invitrogen, USA) was added. Plates were incubated for one hour in the dark. Wells were washed in PBS + 1% BSA, followed by PBS. Fluorescence was read using emission and excitation wavelengths of 490 and 525 nm, respectively, using a GloMax-Multi Detection System plate reader (Promega, USA). Relative quantities of DCT-specific antibodies were calculated as follows: mean fluorescence of wells that received both plasma and the secondary antibody - mean fluorescence of wells that received the secondary antibody only (*i.e*. background fluorescence).

### Statistics

Graphing and statistical analyses were done with GraphPad Prism version 7. Graphs show means and standard errors. Frequencies and numbers of T cells in blood and spleens and plasma-derived antibodies were assessed by one-way analysis of variance with Tukey’s multiple comparison. Numbers of blood-derived cells were normalized based on sample volumes. All numbers were expressed as relative values to account for the inevitable loss of cells during processing and to prevent direct inference of actual physiological numbers. One person processed each set of samples and was blinded to treatment groups. Tukey’s multiple comparison test was applied to two-way analysis of variance for lymph nodes, with anatomical location of nodes and the number of injection sites used as variables. Pearson correlation analysis was used to assess relationships between survival and magnitude of T cell responses or the number of vaccine injection sites and T cell and antibody responses. Differences between treatments were considered significant at p ≤ 0.05.

### Study Approval

All animal-based experiments were approved by the University of Guelph Animal Care Committee (Guelph, Ontario, Canada; animal utilization protocol #1905) and met the requirements of the Canadian Council on Animal Care.

## Electronic supplementary material

Supplementary Information
