# Supplementary material for: Enhancing Immune Responses to Cancer Vaccines Using Multi-Site Injections
Source: Sci Rep. 2017 Aug 16;7:8322. doi: 10.1038/s41598-017-08665-9 (PMC5559552; doi:10.1038/s41598-017-08665-9)
Supplement: Supplementary file 1 — Supplementary Information [file 41598_2017_8665_MOESM1_ESM.pdf]

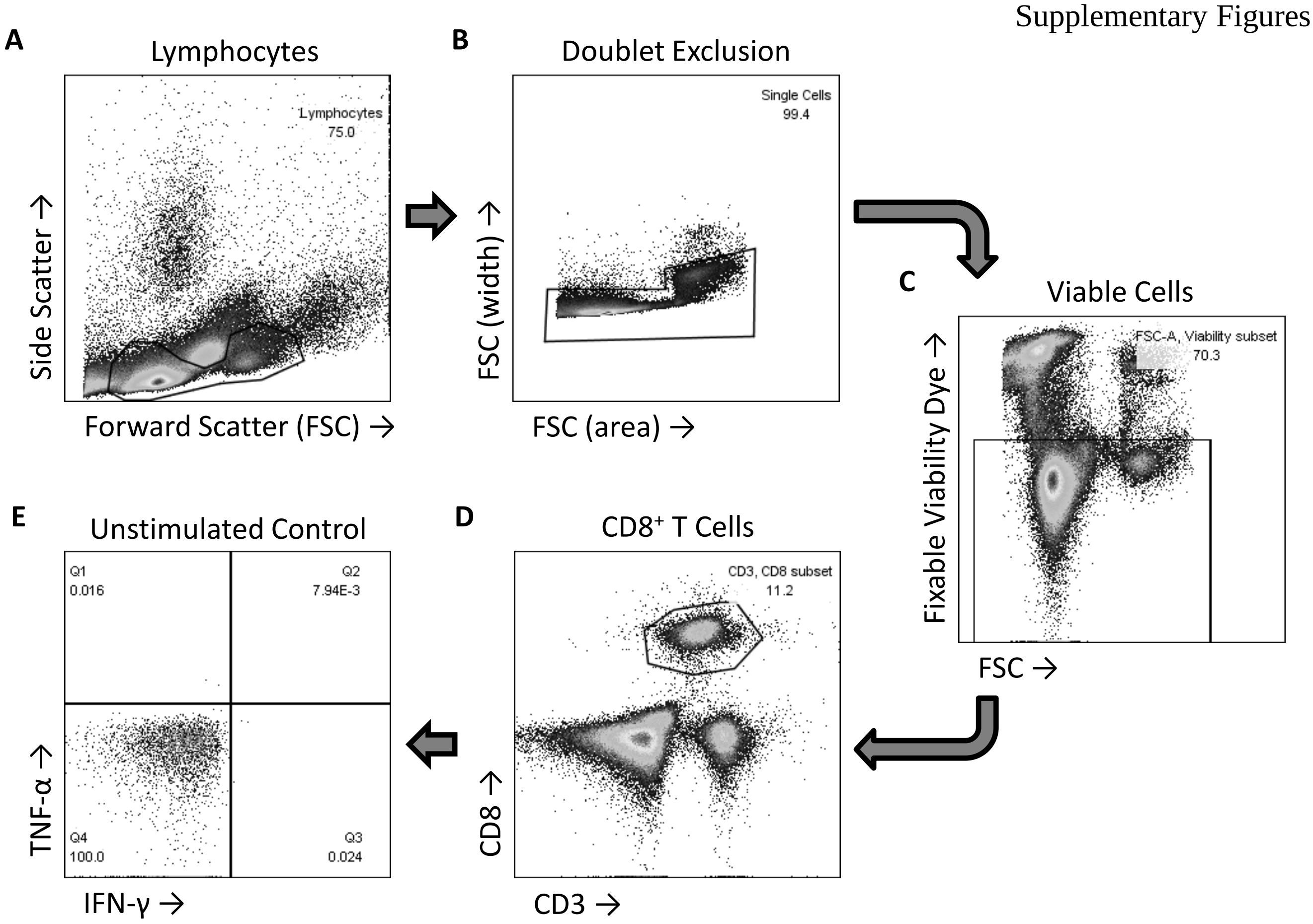

Supplementary Figure 1: Gating strategy for flow cytometric assessment of antigen-specific CD8⁺ T cells. Blood (shown), spleens or lymph nodes from vaccinated or unvaccinated mice were used to assess T cell responses. Erythrocyte-free single-cell suspensions were re-stimulated with peptides representing the immunodominant epitopes of the target antigen. Cells had Fc receptors blocked and were then stained for surface markers and intracellular interferon (IFN)-γ and tumour necrosis factor (TNF)-α. (A) Lymphocytes were gated based on their physical characteristics on a forward scatter (FSC; area) versus side scatter (area) plot. (B) Singlet lymphocytes were then identified on a FSC (area) versus FSC (width) plot. (C) Viable singlet lymphocytes were selected by excluding those that stained with a fixable viability stain. (D) Viable $CD3^+CD8^+$ T cells were subsequently selected for analysis of (E) intracellular cytokine staining to quantify those that were specific for the target peptide (*i.e.* quadrant #2 + quadrant #3). In this example, an typical unstimulated control is shown, for which the background staining of Q2+Q3 was always <0.05%. T cell responses were calculated as: (Q2+Q3 [stimulated]) - (Q2+Q3 [unstimulated]).

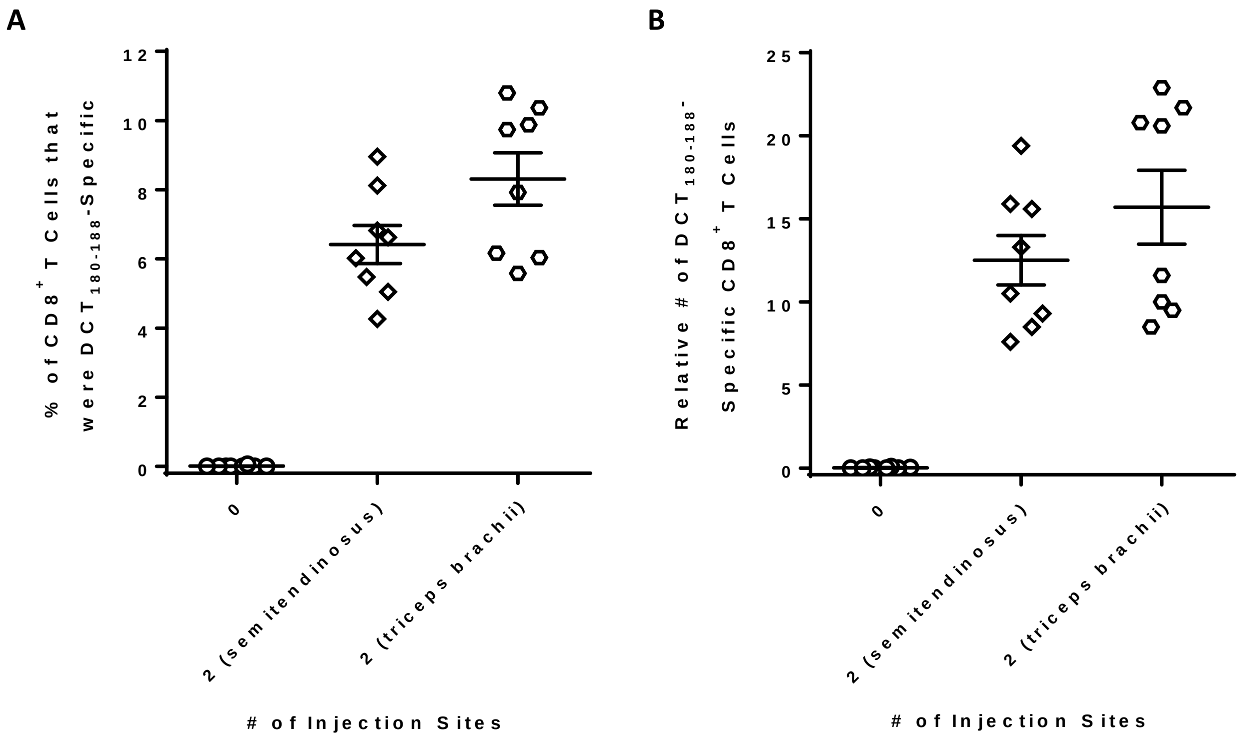

Supplementary Figure 2: Targeting lymphatic regions draining the hamstrings versus triceps brachii with multi-site vaccinations does not alter the magnitude of antigen-specific $CD8^+$ T cell responses. Tumor-free C57BL/6 mice (n = 8 per group; pooled from two experimental replicates) were either unvaccinated (0 injection sites) or vaccinated with a total of $1x10^8$ pfu of a recombinant human serotype 5 adenovirus expressing the melanoma-associated antigen dopachrome tautomerase, with injections spread across two sites (*i.e.* the semitendinosus of both hind limbs versus the triceps brachii of both forelimbs). Fourteen days post-immunization blood-derived $DCT_{180-188}$-specific $CD8^+$ T cells were quantified by flow cytometric detection of intracellular cytokines following *ex vivo* re-stimulation with peptides. The (A) frequency and (B) relative number (normalized based on blood volume) of DCT-specific T cells were assessed by one-way analysis of variance with Tukey's multiple comparison test. Means and standard errors are shown. The anatomical location (*i.e.* hind limbs versus forelimbs) of the multi-site injections did not significantly influence the magnitude of the T cell response ($p>0.05$).

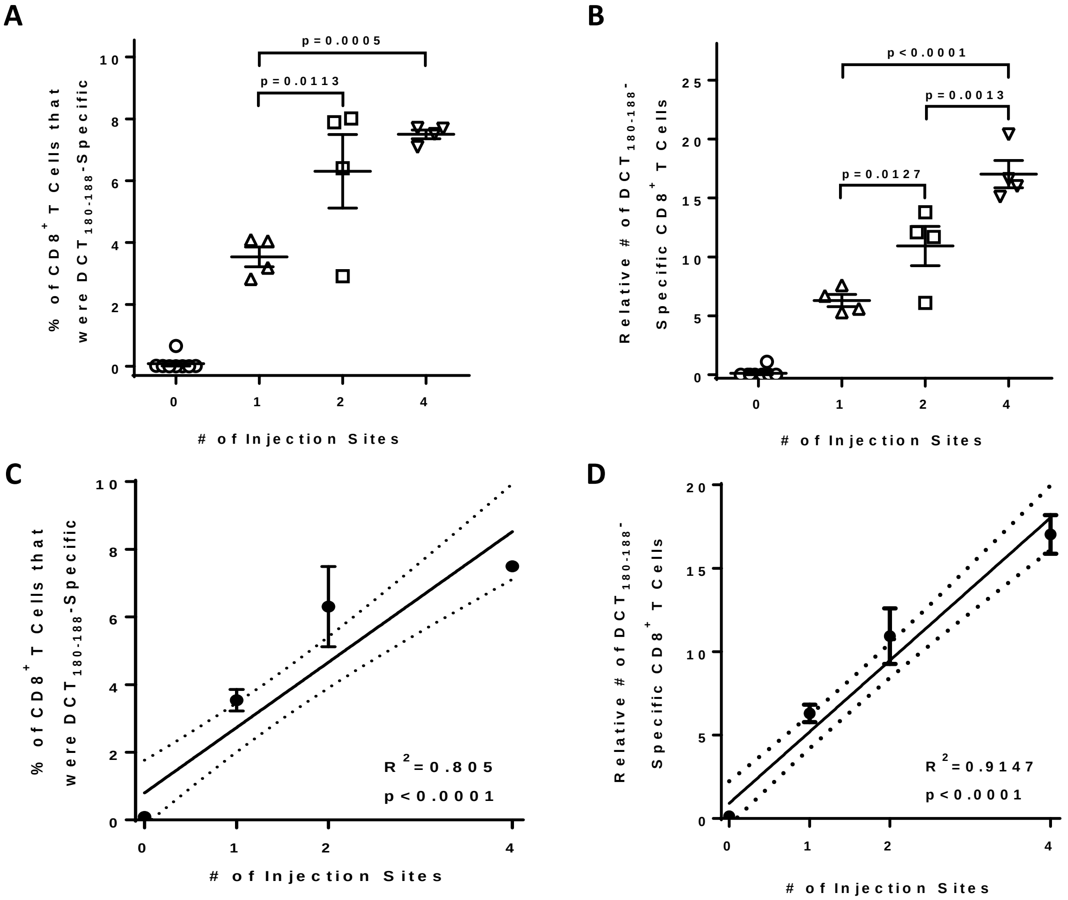

Supplementary Figure 3: The magnitude of blood-derived CD8$^+$ T-cell responses correlates with the number of vaccination sites in tumor-bearing hosts. C57BL/6 mice (n = 4 per group; one experimental replicate) with intradermal B16-F10 melanomas were either unvaccinated (0 injection sites) or vaccinated with a total of 1x10^8 pfu of a recombinant human serotype 5 adenovirus expressing the melanoma-associated antigen dopachrome tautomerase, with injections spread across one (semitendinosus; left hind limb), two (semitendinosus of both hind limbs) or four (semitendinosus of both hind limbs and the triceps brachii of both forelimbs) sites. Fourteen days post-immunization blood-derived DCT$_{180\text{-}188}$-specific CD8$^+$ T cells were quantified by flow cytometric detection of intracellular cytokines following *ex vivo* re-stimulation with peptides. The (A) frequency and (B) relative number (normalized based on blood volume) of DCT-specific T cells were assessed by one-way analysis of variance with Tukey's multiple comparison test. Strong, significant Pearson correlations were found between (C) frequency and (D) relative number of DCT-specific T cells and the number of vaccine injection sites. Means and standard errors are shown.

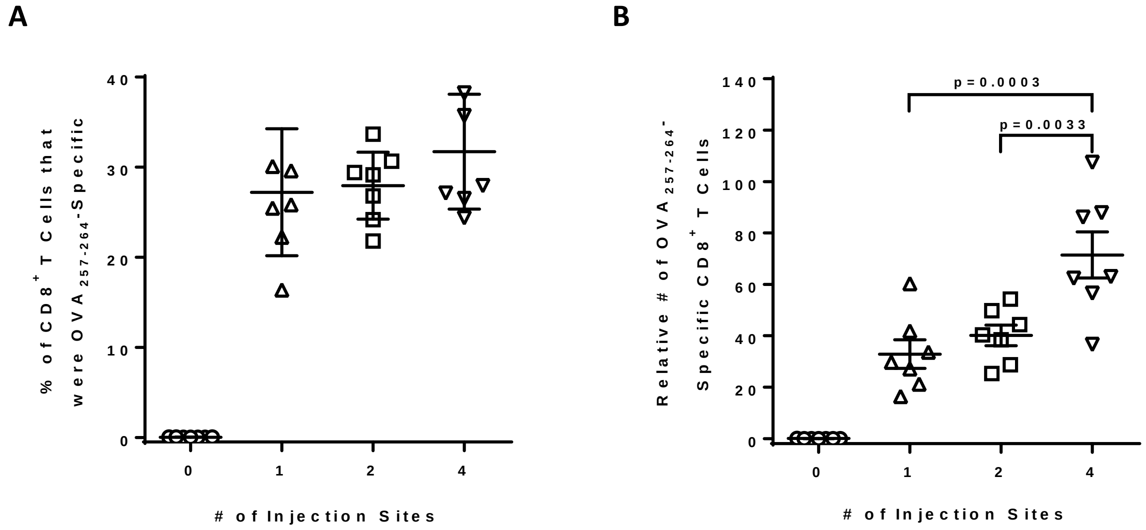

Supplementary Figure 4: Multi-site vaccination can enhance the magnitude of T-cell responses to a foreign antigen. Tumor-free C57BL/6 mice (n = 7 per group; pooled from two experimental replicates) were either unvaccinated (0 injection sites) or vaccinated with a total of $1x10^8$ pfu of a recombinant human serotype 5 adenovirus expressing the immunodominant epitope from chicken ovalbumin ($OVA_{257-264}$), with injections spread across one (semitendinosus; left hind limb), two (semitendinosus of both hind limbs) or four (semitendinosus of both hind limbs and the triceps brachii of both forelimbs) sites. Fourteen days post-immunization blood-derived $OVA_{257-264}$-specific $CD8^+$ T cells were quantified by flow cytometric detection of intracellular cytokines following *ex vivo* re-stimulation with peptides. The (A) frequency and (B) relative number (normalized based on blood volume) of DCT-specific T cells were assessed by one-way analysis of variance with Tukey's multiple comparison test. Means and standard errors are shown. Four-site vaccination was superior to both one- and two-site protocols with respect to the number of OVA-specific T cells that were induced.
